# The double task of preventing malnutrition and overweight: a quasi-experimental community-based trial

**DOI:** 10.1186/1471-2458-13-212

**Published:** 2013-03-09

**Authors:** José I Navarro, Dirce M Sigulem, Alexandre A Ferraro, Juan J Polanco, Aluísio JD Barros

**Affiliations:** 1 Nutrition Post Graduation Program, Federal University of São Paulo, São Paulo, SP, Brazil; 2 Paediatrics Department, Medical School of the University of São Paulo, São Paulo, SP, Brazil; 3 Social and Demographic Studies Center, Santo Domingo, Dominican Republic; 4 Postgraduate Program in Epidemiology, Federal University of Pelotas, Pelotas, RS, Brazil

**Keywords:** Malnutrition, Overweight, Obesity, Infant, Integrated management of childhood illness, Community health workers, Program evaluation, Nutritional transition

## Abstract

**Background:**

The Maternal-Child Pastoral is a volunteer-based community organization of the Dominican Republic that works with families to improve child survival and development. A program that promotes key practices of maternal and child care through meetings with pregnant women and home visits to promote child growth and development was designed and implemented. This study aims to evaluate the impact of the program on nutritional status indicators of children in the first two years of age.

**Methods:**

A quasi-experimental design was used, with groups paired according to a socioeconomic index, comparing eight geographical areas of intervention with eight control areas. The intervention was carried out by lay health volunteers. Mothers in the intervention areas received home visits each month and participated in a group activity held biweekly during pregnancy and monthly after birth. The primary outcomes were length and body mass index for age. Statistical analyses were based on linear and logistic regression models.

**Results:**

196 children in the intervention group and 263 in the control group were evaluated. The intervention did not show statistically significant effects on length, but point estimates found were in the desired direction: mean difference 0.21 (95%CI −0.02; 0.44) for length-for-age Z-score and OR 0.50 (95%CI 0.22; 1.10) for stunting. Significant reductions of BMI-for-age Z-score (−0.31, 95%CI −0.49; -0.12) and of BMI-for-age > 85^th^ percentile (0.43, 95%CI 0.23; 0.77) were observed. The intervention showed positive effects in some indicators of intermediary factors such as growth monitoring, health promotion activities, micronutrient supplementation, exclusive breastfeeding and complementary feeding.

**Conclusions:**

Despite finding effect measures pointing to effects in the desired direction related to malnutrition, we could only detect a reduction in the risk of overweight attributable to the intervention. The findings related to obesity prevention may be of interest in the context of the nutritional transition. Given the size of this study, the results are encouraging and we believe a larger study is warranted.

## Background

In 2008, a group of researchers produced a series of articles drawing the attention of national policymakers and the international community to put in place and maintain effective programs to fight mother and child undernutrition among the priorities for achieving several of the Millennium Development Goals [1]. It is estimated that the joint effect of the main indicators of child undernutrition and sub-optimum breastfeeding practices accounts for 35% of the deaths of children under 5 years of age worldwide [2]. Among the survivors, important sequelae remain; the long term consequences most strongly associated to undernutrition during the first years of age include: shorter adult height, less schooling, reduced economic productivity, and lower offspring birth weight in women [3]. The analysis of representative data from less economically developed countries shows that the phenomenon of growth faltering takes place during the first years of life, and then it stabilizes [4]. The nutritional recovery of children who were malnourished during their first years of life fails to avoid important consequences, and a fast weight gain of these children in the following years can increase the risk of cardiovascular and metabolic diseases in adulthood [2]. It is therefore clearly necessary to implement programs aimed at preventing undernutrition since pregnancy and during the child’s first years of life.

During the past few decades important progress has been made in the reduction of child undernutrition in developing countries. In Latin America and the Caribbean, the prevalence of stunting dropped by 44% between 1980 and 2000 [5]. Nevertheless, this progress has been very unequal both at the regional level and within each country, as child undernutrition persists, although it is a high priority. On the other hand, as the fight against undernutrition advances, the problem of obesity emerges at a quite accelerated pace [6]. For example, in the Dominican Republic, although the goal of halving low weight-for-age among children under 5 was quickly achieved (10.4% in 1991 vs 5.3% in 2002) [7,8], the prevalence of weight-for-height greater than +2 standard deviations increased from 2.8% in 1991 to 6.5% in 2002 [9]. It is estimated that the prevalence of obesity among adults in this country will increase from 16.9% in 2002 to 30.5% by 2015 [10].

Thus, it is urgent to develop large-scale interventions addressed at promoting a more healthy nutritional transition in low-income and middle-income countries, reducing undernutrition while preventing the increase of incidence of chronic diseases related to the excess of weight [1].

Systematic reviews have detected the positive effect of community lay health workers in promoting child health practices such as immunization and breastfeeding, however they did not find evidence of an effect on nutritional status [11,12]. Studies on the impact of a volunteer community health worker program (Child Pastoral Program) in Brazil did not find effect on stunting [13,14]. Nonetheless some of the intervention on complementary feeding practices delivered to mothers by community workers have had positive effect on infant growth [15,16].

In the Dominican Republic, the Maternal-Child Pastoral, a volunteer community organization, has designed a community intervention to improve integrated health and development during early childhood. The previous experience of the Brazilian Child Pastoral Program was adapted and the following actions have been added to improve the effect on child’s nutritional status: to start the intervention during pregnancy; to create guidelines for mother group meetings; to create guidelines to help nutritional counseling that take into account the trends in child’s growth curve; to deliver educative material to mothers about complementary feeding; to counsel about iron and vitamin A supplementation, including the dispensation of supplements in case local health facilities had not done before.

Among the objectives of the intervention are the reduction of both malnutrition and the risk of overweight in the first two years of age. This intervention was first implemented as a pilot in mid 2004 in coordination with the local offices of the Pan-American Health Organization (PAHO/WHO), the United Nations Children’s Fund (UNICEF), the Ministry of Public Health of the Dominican Republic, and the National Breastfeeding Commission within the family and community component of the Integrated Management of Childhood Illness (IMCI) strategy. This study tests the hypothesis that children participating in the Mother-Child Pastoral from pregnancy would have better nutritional indicators in respect both to stunting and overweight, in the second year of age, compared to children in communities of similar socioeconomic conditions where the program does not exist.

## Methods

### The intervention

According to the causal model proposed by UNICEF on the factors affecting child survival, growth, and development [17], this intervention acts on an intermediary level by means of an educative process which tries to modify mother and child care practices. Taking as a reference the key family practices of the IMCI [18,19] a strategic decision was made to start the intervention from the key practice that aims an adequate antenatal care. The first step was to create groups of pregnant women which met every fifteen days according to protocols defined in ten educational meetings on health and nutrition during pregnancy. Simultaneously to these meetings were held monthly home visits. Fortnightly home visits were carried out during the first month and a half after child birth in order to support breastfeeding and newborn care. Thereafter, group meetings and semi-structured home visits were carried out once every month to deal with breastfeeding, vaccination, newborn care, danger signs, complementary feeding, micronutrient supplementation, prevention and treatment of infectious diseases, growth monitoring, early stimulation, and prevention of accidents.

The educational process was based on a concept of transformational education similar to the decision-making process known as Triple A (*assessment-analysis-action*) proposed by UNICEF and WHO [17]. The education agents are community volunteers known as community counselors (most of whom are women with the same formal education profile as the mothers benefited), who participate in a 60-hour basic training facilitated by health professionals previously trained in key practices of the IMCI community component.

As part of the intervention, the children’s physical growth was monitored at the monthly meetings of mothers (or during the home visits) to check the progress on their weight-for-age curve. The children were weighed by the community counselors using spring scales provided by the UNICEF Supply Division. The measures were plotted along the NCHS growth charts for assessing the trend of the child’s weight-for-age curve. Based on this evaluation, the counselors followed an advice protocol called *Nutritional Advice Based on the Growth Curve.* This protocol included recommendations to enhance caloric density, frequency and/or quantity of food in the case that the trend of the curve showed insufficient weight gain and revision of inadequate practices of increased energetic ingestion in the event that the growth curve increased at a pace that led to overweight for age. When the deflection from the expected trend continued for more than two consecutive evaluations, the counselor referred the mother for evaluation by a health professional, and then resumed monitoring. The education materials were prepared based on reference documents and materials published by WHO/PAHO [20,21] and the volunteer’s manual of the Child’s Pastoral in Brazil [22].

### Study design

A paired quasi-experimental study was carried out with 8 intervention geographic areas and 8 control ones with similar socio-economic and cultural characteristics. Sampling was not random, since intervention areas had been decided before this study. Each area, named here “branch”, was made of communities assisted by a catholic parish. Control branches were selected following the advice of an urbanist and an anthropologist together with the assessment of a wealth index used by demographic and health surveys [23]. National Census of Population and Households of 2002 was the source of this information. Paired branches were similar (Table 1). Health authorities were interviewed to assess possible differences in access to health facilities, with no difference found. Excluding the existence of usual local health facilities, in both groups there were no other community health and nutrition intervention for pregnant women and children under 2 years of age.

**Table 1:** Community average wealth index in the study groups, according to the National Census 2002

	**Intervention group**			**Control group**	
**Branch**		**Average**	**Branch**		**Average**
La Ciénaga		45.1	La Zurza		42.4
Los Guandules		46.2	Gualey		47.4
Guachupita		57.5	24 de Abril		59.0
El Manguito		65.5	Los Praditos		61.4
Municipio Consuelo		52.3	Municipio Quisqueya		45.6
Bateyes Consuelo		35.3	Bateyes Santa Fe		39.7
Parajes Ntra. Sra. de la Paz		54.1	Parajes Ntra. Sra. Consolación		49.9
Brisas de los Palmares		(…)^a^	Barrio Nuevo		(…)^a^

^a^ The maps and codes for these communities could not be gathered.

**Figure 1:**
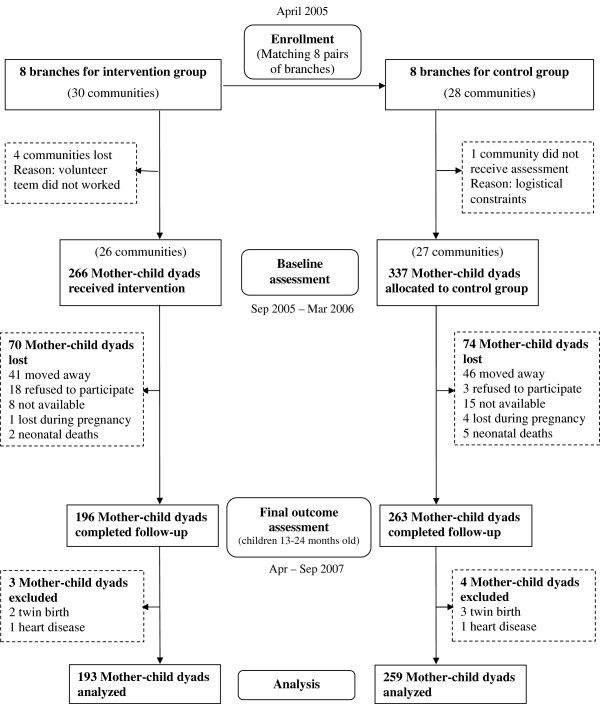
Flow of study participants.

The intervention started in April 2005 with pregnant women (before the third quarter of pregnancy) living in the 8 branches defined as intervention group (Figure 1). Baseline data were collected in the last quarter of pregnancy or in the four months immediately after child birth, both in the intervention group and in the control group. When the mothers were interviewed during pregnancy, the interviewers visited them again during the first four months immediately after child birth to collect data related to pregnancy outcomes. The reason for using these two specific biological periods was to obtain a greater number of samples in a given time span and collect data during pregnancy in a sub-group, for another study. Data on health and nutrition indicators were collected in both groups when the children were aged between 13 and 24 months (April-September 2007). Children from multiple births or with congenital chronic diseases were excluded from the study.

### Variables studied

**Figure 2:**
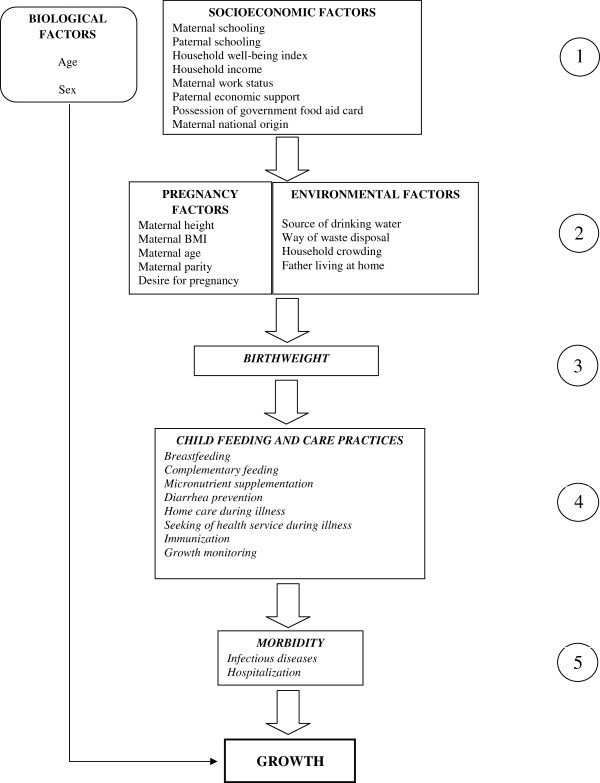
Conceptual model of factors related to child growth.

Figure 2 shows the conceptual model built for this study. In the selection of indicators for the variables studied and in the formulation of questionnaires, the indicators used in several surveys and evaluation studies were taken into account [8,24-27].

The primary outcome indicators selected for this study were: mean length-for-age *Z-*score (LAZ), prevalence of stunting (LAZ < −2), mean BMI-for-age *Z-*score (BAZ) and prevalence of BMI-for-age > 85^th^ percentile, defined as “at risk for overweight.”

### Sample size

The sample size was calculated for a one-tailed test considering that the intervention could either improve the health and nutrition indicators or have no effect on them, but it could not worsen them [28]. The confidence level established was 95% and the statistical power 80%. With the information available at the time of the intervention design [29], and with data previously collected in a few communities in which the intervention was going to be conducted, a 20% for stunting was taken as reference for the child population aged 12–23 months. With a sample size of 283 in each group (intervention-control) it would be possible to detect reductions by 40% in the prevalence of stunting. Considering 10% of loss during monitoring, 312 cases would be allocated in each group.

### Data collection

The questionnaires were applied by medical students, as part of a course in epidemiology, and the anthropometrists were previously standardized [30]. For practical reasons it was not feasible to conduct a double-blind study.

Databases were prepared using Epi Info, version 3.2 for Windows (Center for Disease Control and Prevention, Atlanta, GA, USA). After a double-entry procedure, typing errors were identified and corrected using the Epi Info “Data Compare” module. Later revisions for amplitude and consistency were administered.

For anthropometric measurements, mother/child electronic scales with a precision of 0.1 Kg (UNISCALE, UNICEF Supply Division) and Shorr length measuring boards accurate to 0.1 cm (Shorr Productions Growth Unlimited, Olney, Md., USA) were used. Measurements were made according to procedures recommended by the World Health Organization [31,32], and the average measure of two standardized anthropometrists was used, repeating up to twice whenever the difference between both was over 0.7 cm or 0.1 Kg [33]. The anthropometric indexes were calculated by WHO Anthro 2005 software, using the new WHO Child Growth Standards.

### Statistical analysis

Since the intervention was allocated according to geographic areas, multilevel analysis were conducted to evaluate the presence of nested random effects on the main results, taking into consideration the interdependency between children from a same branch and a same community. However, for the regression models of the primary outcomes LAZ, LAZ < −2, BAZ and BMI-for-age > 85^th^ percentile, at the final assessment, there was no significant intraclass correlation and the multilevel models were not different from the traditional ones when compared through the likelihood ratio test. Therefore, it was decided that the analysis would be made without resorting to the multilevel model techniques. Two-tailed tests were used.

Baseline data between the study groups were compared using chi-square test for categorical variables and t test for continuous. In assessing the effect of the intervention on the outcomes, multivariate linear regression models were performed for adjustment in relation to all potential confounding factors that had p<0.10 in the model.

All of these analyses were conducted with Stata, version 10.0 (Stata Corp. LP, College Station, TX, USA).

### Loss of follow-up

Given the high mobility of the studied population it was not possible to measure the outcome variables in those subjects that were lost during the study. To assess whether these individuals differed from those who were followed up to the end, statistical analyses were carried out comparing baseline data.

### Ethical aspects

The study was approved by the Committee of Ethics in Research of the Federal University of São Paulo and was implemented in coordination with the Ministry of Public Health of the Dominican Republic. Mothers were asked to sign a written consent. In both intervention and control groups malnourished children were referred to the specific health service.

## Results

### Sample

Figure 1 shows the flow of participants through the study [34]. After about a year and a half of follow-up the intervention group lost 26% of its subjects and the control group lost 22%. The main cause for this was changing the place residence. The number of analyzed subjects was 193 in the intervention group and 259 in the control group. After comparing 16 baseline variables between the lost-of-follow-up subjects and the completely-followed-up ones (see Additional file 1), it was found that within the intervention group lost-of-follow-up mothers were younger (21.3 vs 24.1 years) and their children were more frequently first born (42.5% vs 28.5%). Within the control group lost-of-followed-up mothers had a lower household wealth index (43.01 vs 49.43) and more frequently disposed waste in a nearby dump, river or glen (48.5% vs 33.9%).

Table 2 presents the baseline data homogeneity analysis of both groups. From the 21 studied variables three had a p<0.05 (child’s age, maternal work status and waste disposal) and two p ≅ 0.1 (household income and maternal age). These variables were included in the multivariate regression analysis to adjust for a possible confounding effect.

**Table 2:** Baseline data according to study group

	**Control group**	**Intervention group**	
	**n= 259**	**n= 193**	
**Characteristics**	**Frequency (%)**^**a**^	**Frequency (%)**^**a**^	***P***
Final child’s age, months ^b c^	19.74 ± 2.08	20.32 ± 1.70	0.002*
Sex of the child			0.373
Male	134 (51.74)	108 (55.96)	
Female	125 (48.26)	85 (44.04)	
Maternal education, years ^b^			0.805
0-5	56 (22.05)	44 (23.04)	
≥ 6	198 (77.95)	147 (76.96)	
Paternal education, years ^b^			0.794
0-5	88 (36.51)	66 (35.29)	
≥ 6	153 (63.49)	121 (64.71)	
Household wealth index ^c^	49.43 ± 20.55	50.54 ± 19.14	0.559
Household income, minimum wage per month			0.123*
1	201 (80.40)	138 (74.19)	
≥ 2	49 (19.60)	48 (25.81)	
Mother had worked in the last 12 months ^b d^	86 (33.59)	88 (45.83)	0.009*
Permanent economic support from father ^b^	193 (75.39)	152 (79.58)	0.296
Mother possesses “Comer es primero” card ^b e^	38 (14.84)	32 (16.67)	0.599
National origin of the mother			0.819
Dominican	230 (89.49)	174 (90.16)	
Haitian	27 (10.51)	19 (9.84)	
Piped water inside the house (or outside, rural)	114 (44.02)	78 (40.41)	0.444
Waste disposed in a nearby dump, river or glen	80 (33.90)	42 (23.33)	0.019*
More than 2 children aged 0–4 in the household	30 (11.58)	19 (9.84)	0.557
Father lives in the house with mother ^b^	171 (66.80)	133 (69.27)	0.579
Maternal height, cm ^c^	159.12 ± 6.43	159.03 ± 6.06	0.894
Maternal BMI, kg/m^2 f^	22.46 ± 1.20	22.27 ± 1.20	0.648
Maternal age, years ^f^	23.28 ± 1.26	24.08 ± 1.26	0.125*
First born child	71 (27.41)	55 (28.50)	0.799
Desire for pregnancy			0.702
Wanted to become pregnant at the time	89 (34.36)	63 (32.64)	
Wanted to wait or did not want more children	170 (65.64)	130 (67.36)	
Birthweight, kg ^c^	3.20 ± 0.59	3.18 ± 0.55	0.742
Low birthweight (< 2.5 kg)	19 (7.36)	18 (9.38)	0.443

* Variables with p<0.20 (to take into account in the regression models).

^a^ The percentages are based on the number of completed responses for each particular variable.

^b^ This variable was also measured in the 2007 questionnaire, and those data were analyzed here.

^c^ The values presented are: average ± standard deviation.

^d^ Has worked outside of the home or in the home making money.

^e^ This is the principal food aid program of the government. It transfers money to poor families to purchase food. This variable has been included because of its potential impact on child growth.

^f^ The presented values are: geometric mean ± geometric standard deviation, because the logarithmic transformation of the variable was necessary.

### Anthropometric indicators

The results of the anthropometric indicators revealed a low prevalence of wasting (weight-for-length *Z-*score < −2) both in the control group and in the intervention group, respectively 1.16% and 2.07%, with no significant difference (*P*=0.436). These prevalence values are within the expected range in the WHO standard population and are close to the findings of the 2007 Dominican Republic Demographic and Health Survey [35] (2.2% for children younger than five). In the same survey, the prevalence of low weight-for-age in children under 5 was 3.1%; in our study it was 3.86% in the control group and 2.07% in the intervention group, a difference that lacks statistical significance (*P*=0.278).

The results on length were not statistically significant (Table 3). The mean length-for-age *Z-*score (LAZ) was −0.66 in the control group and −0.46 in the intervention group, resulting in an adjusted difference of 0.21 (*P* = 0.067). The prevalence of stunting was 11.97% in the control group and 7.25% in the intervention group, with an adjusted odds ratio of 0.50 (*P* = 0.085). On the other hand, an analysis of LAZ < −1showed a prevalence of 36.29% in the control group and 33.16% in the intervention group (*P*=0.490). No significant interaction effects were found.

**Table 3:** Effect of the intervention on anthropometric indicators

** Anthropometric indicator**	**Control group**	**Intervention group**	**Crude effect**		**Adjusted effect**	
	**n = 259**	**n = 193**				
	***x ± SD***	***x ± SD***	Δ ***x (CI 95%)***	***P***	Δ ***x (CI 95%)***	***P***
Length-for-age (Z score) ^a^	−0.66 ± 1.09	−0.46 ± 1.21	0.20 (−0.01; 0.41)	0.067	0.21 (−0.02; 0.44)	0.067
BMI-for-age (Z score) ^b^	0.35 ± 0.92	0.13 ± 0.94	−0.22 (−0.40; -0.05)	0.012	−0.31 (−0.49; -0.12)	0.001
	*Freq (%)*	*Freq (%)*	*OR (95% CI)*	*P*	*OR (95% CI)*	*P*
Stunting (LAZ < −2) ^c^	31 (11.97)	14 (7.25)	0.58 (0.30; 1.11)	0.101	0.50 (0.22; 1.10)	0.085
Risk of overweight (BMI-for age > 85th percentile)^d^	58 (22.39)	30 (15.54)	0.64 (0.39; 1.04)	0.070	0.43 (0.23; 0.77)	0.005

^a^ Adjusted effect for: age, sex, maternal work status, household wealth index, number of children in the home, maternal height, maternal BMI and birthweight.

^b^ Adjusted effect for: age, sex, maternal height, maternal BMI and birthweight.

^c^ Adjusted effect for: age, sex, maternal education, maternal height and maternal BMI.

^d^ Adjusted effect for: age, sex, maternal BMI and birth weight.

The mean BMI-for-age *Z-*score was 35 ± 0.92 in the control group and 0.13 ± 0.94 in the intervention group, with an adjusted effect of −0.31 (*P=*0.001). The categorical analysis of this variable found a difference in the proportion of children with BMI-for-age > 85^th^ percentile, with values of 22.39% in the control group and 15.54% in the intervention group, with an adjusted odds ratio of 0.43 and confidence interval between 0.23 and 0.77 (*P*=0.005). To obtain the figures of this effect in terms of prevalence ratio, the same model was applied using Poisson regression with robust variance [36], which led to a prevalence ratio of 0.52, indicating a reduction by 48% in the proportion of children with risk of overweight. On the other hand, when the *Z-*score with cut-off point +2 was considered instead of 85^th^ percentile, the resulting prevalence was 3.47% in the control group and 1.55% in the intervention group (*P*=0.209).

### Intermediary factors

Table 4 shows the results of several intermediary factors that conceptually could be in the pathway between the intervention and the outcomes of the study. In the intervention group, the growth monitoring activities, home health promotion visits, and participation of mothers in any of the group meetings on child health and nutrition were notably higher than in the control group. While in the control group the proportion of mothers who had participated in any child health and nutrition meeting since the child was born was 13%, in the intervention group it was 98%. When asking mothers of the intervention group to recall the number of encounters that they participated in since the birth of their child, the proportion with participation in six or more meetings was 68% and in three or more meetings 93%.

**Table 4:** Intermediary factors – health promotion, feeding practices and disease prevention and treatment

	**Control group**	**Intervention group**		
**Indicator of the intermediary factor**	**n = 259**	**n = 193**	**Adjusted effect**^**b**^	
	**Freq (%)**^**a**^	**Freq (%)**^**a**^	***OR (95% CI)***	***P***
*Health promotion*				
Child was weighed in the last 4 months	131 (51.37)	159 (83.25)	4.64 (2.95; 7.32)	< 0.001
The child growth curve was explained to the mother	69 (27.71)	136 (71.20)	6.45 (4.24; 9.80)	< 0.001
Mother received counseling on infant and young child feeding practices in the last 4 months	47 (18.36)	134 (70.16)	11.19 (7.02; 17.82)	< 0.001
Mother received a home visit from a community health agent or health professional in the last 4 months	30 (12.00)	132 (69.11)	16.41 (10.06; 26.77)	< 0.001
Mother participated in child health and nutrition meeting since the child’s birth	33 (13.10)	188 (97.92)	311.9 (108.5; 896.5)	< 0.001
*Feeding practices*				
Mother took a Vitamin A capsule in the 8 weeks following birth	51 (20.73)	126 (68.85)	8.45 (5.45; 13.11)	< 0.001
Exclusive breastfeeding through 6 months of age	6 (2.34)	14 (7.29)	3.28 (1.24; 8.69)	0.017
Predominant breastfeeding through 6 months of age ^c^	13 (5.08)	31 (16.15)	3.60 (1.83; 7.09)	< 0.001
Child currently breastfeeding	67 (26.17)	50 (26.04)	0.97 (0.63; 1.49)	0.885
Child received 5 or more feedings with solid or semisolid foods in the last 24 hours	48 (18.53)	53 (27.46)	1.62 (1.03; 2.55)	0.037
Child consumed foods rich in vitamin A in the last 24 hours ^d^	66 (25.48)	46 (23.83)	0.92 (0.59; 1.41)	0.688
Child consumed fruits in the last 24 hours	146 (56.37)	129 (66.84)	1.52 (1.02; 2.26)	0.039
Child received vitamin A supplement	171 (66.80)	159 (82.81)	2.35 (1.49; 3.71)	< 0.001
Child received iron supplement in the last 3 months	109 (43.43)	165 (86.39)	8.23 (5.07; 13.37)	< 0.001
*Disease prevention and treatment*				
Child consumed water from the faucet, not boiled, without chlorine	50 (19.53)	21 (10.94)	0.48 (0.28; 0.85)	0.011
with greatest frequency				--
Child used bottle in the last 24 hours	198 (77.34)	140 (72.92)	0.79 (0.51; 1.23)	0.298
Child had diarrhea in the last 2 weeks	41 (16.02)	29 (15.10)	0.99 (0.59; 1.67)	0.980
Child received more liquids during diarrhea	25 (58.14)	24 (77.42)	3.42 (1.06; 11.07)	0.040
Child received ORS during diarrhea	28 (63.64)	27 (87.10)	3.86 (1.14; 13.02)	0.030
Child received equal or greater amount of foods during diarrhea	23 (53.49)	14 (45.16)	0.63 (0.24; 1.66)	0.347
Mother mentioned a sign of emergency during diarrhea	170 (65.64)	147 (76.17)	1.67 (1.10; 2.54)	0.016
Child completed vaccination schedule in the first year of life	90 (35.16)	100 (52.08)	2.00 (1.37; 2.94)	< 0.001
Child was hospitalized in the last 12 months	60 (23.44)	48 (25.00)	1.09 (0.70; 1.68)	0.702

^a^ The percentages are based on the number of completed responses for each particular variable.

^b^ Adjusted for household income and maternal education, work status, age and national origin when any of these covariates had p<0.10 in the multivariate regression analyses.

^c^ Predominantly breast milk, but may also have received water, tea, and fruit juice.

^d^ Foods rich in vitamin A: carrot, pumpkin, sweet potato and liver.

The results related to some of the feeding practices show a higher consumption of vitamin A and iron supplements by children and mothers in the intervention group. When mothers were asked about the age that child started receiving regularly the food items from a list, the prevalence of exclusive breastfeeding was shown to be 2.34% in the control group and 7.29% in the intervention group (*P*=0.017). On the other hand, in the intervention group there was a higher proportion of children that consumed five or more solid or semisolid food items over the past 24 hours (27.46% vs 18.53% in the control group, *P*=0.037). However, there was no difference either in the consumption of vitamin A rich food or in most of the particular food items in the previous 24 hours or in the previous 7 days.

Table 4 also shows the results of some factors related to the prevention and treatment of illness. Differences were found that indicate more adequate practices in the intervention group related to immunization, drinking water and administration of liquids and oral rehydration salts during diarrhea. However, there was no difference between the groups with regard to the use of bottle and the amount of food consumed during diarrhea. Neither were there any differences between the groups with regard to the prevalence of diarrhea over the past two weeks or in the proportion of children hospitalized during the past 12 months.

## Discussion

The results of this study show that the intervention was associated with a lower risk of overweight. The approximation of the mean Z score of this index towards the value corresponding to the WHO reference population (Z = 0), with a decrease in the proportion of children with a score above the 85th percentile without causing an increase in wasting, points to a healthy decrease in the risk of overweight. The size of the adjusted effect (OR = 0.43) is equivalent to a decrease by nearly 48% in relation to the prevalence in the control group. This effect is important under the preventive point of view; children with body mass index over 85th percentile during early childhood have a high probability of being overweight in adolescence [37,38].

Since the study aimed to analyze not only the effect on nutritional indicators but also on various health aspects that the intervention intended to modify (within the integrated approach of the IMCI), no data were collected on some intermediary factors that could have explained better the causal pathway of the effects on overweight. However, the results observed in the activities related to growth monitoring, coupled with the fact that the nutritional counseling protocols included clear instructions about what issues should be discussed with the mother when the weight-for-age curve had a tendency towards the overweight-for-age zone, support the plausibility of attributing these results to the intervention.

As expected in impoverished communities, the mean length-for-age *Z-*score (LAZ) in both groups was below the corresponding level at the WHO reference population. The intervention did not show statistically significant effects on length, but point estimates found were in the desired direction, with a mean difference of 0.21, which is similar to that found in other educational interventions. In a recent review study carried out by Imdad et al. [39], eight studies were selected that evaluated interventions based on maternal education regarding complementary feeding, without the provision of food, resulting in a positive effect in linear growth with a weighted mean difference of 0.21.

A limitation on the probabilistic analysis of the effect of the intervention on the prevalence of stunting was the sample size. It was calculated for a one-tailed test based on an expected prevalence of 20% in the control group, while the found value was 12%. For the magnitude of the intervention effect that was found, it would have required a larger sample size for a significance level of 95%. Furthermore, the sample size calculation did not take into account the clustering.

Our study found positive effect in several indicators of intermediary factors. Whereas in the control group there was a gradual decrease in action regarding growth monitoring, falling from 51% of children weighed in the past 4 months to 28% in cases where an explanation was given to the mother about the results of the curve, up to 18% receiving feeding advice, the intervention group showed another profile: 83% of the children had been weighed and approximately 70% of the mothers had received both explanations about the growth curve and child feeding advice. The intervention performed by the lay health volunteers showed also significant effects in micronutrient supplementation. The proportion of children supplemented with vitamin A capsules and with ferrous sulfate drops was higher in the intervention group, thus resulting in a lower prevalence of anemia, with adjusted odds ratio of 0.57 (*P*=0.011) for Hb < 11.0 g/dl and 0.46 (*P*<0.001) for Hb < 10.0 g/dl (article in preparation). As we expected, effects on food consumption were not as positive as those on micronutrients consumption. Nevertheless there was an increase in exclusive breastfeeding and in the proportion of children who had consumed 5 or more solid food portions in the last 24 hours.

Upon reviewing the eight studies analyzed by Imdad et al. [39], we observed that none of them evaluated the possible effect of the intervention with regard to overweight. In the context of nutritional transition in low-income and middle-income countries, we can consider that the principal contribution of our study is to integrate the prevention of children becoming overweight in an intervention program focused primarily on preventing malnutrition. Furthermore, in current scientific literature regarding interventions where the primary goal is the prevention of childhood obesity, there are very few intervention studies that focus on the first two years of age [40]. In a recent systematic review about interventions to prevent obesity among children aged 0–5 years, seven of twenty-five studies had included younger than 2 years of age, but just two had reported anthropometric measurements, one of them finding a positive trend towards overweight prevention [41]. Another contribution from this present study is that its results suggest that in situations in which body-mass-index is difficult to be periodically assessed early intervention to prevent overweight can be based on monitoring the weight-for-age.

The outcomes were assessed against the WHO Child Growth Standards, while during the intervention the mothers were given feeding advice based on plotting the child’s growth against NCHS weight-for-age charts due to the fact that until then the new standards had not been adopted. As the average weight of infants included in the WHO standards was above the NCHS median during their first six months of life, and thereafter continued below [42], we can deduce that during the intervention there was a tendency to be less demanding in controlling risk of underweight in the first six months (when the NCHS references identify lower prevalence of underweight than WHO standards) and be more demanding in this matter after seven months (when NCHS references identify higher prevalence of underweight than WHO standards). Opposite trends would be present in regards to control the risk of higher than expected weight for age. We could think that these tendencies due to differences between the WHO standards and NCHS references were diminished during the intervention by the fact that counseling protocols oriented to act not only when achieving the cutoffs level of underweight or high weight for age, but also by observing early trends of diversion of the curve.

Among the limitations of this study is included the fact that we did not use an intention to treat analysis, due to difficulties to gather the outcome data in the missing cases. This weakens its external validity in regards to public health policies, as their conclusions are applicable on the condition of the families remaining in the intervention process. Loss of follow-up after allocation is a frequent limitation in public health program studies. In this research, women who dropped out differed from those who remained in the study in 2 of 16 baseline variables analyzed in the intervention group, and in 2 variables in the control group. These differences did not negatively affect the homogeneity of the study groups among the remaining cases.

Furthermore, although important intervention performance indicators were used regarding the last four months of the intervention, it would have been important to evaluate some other longitudinal indicators besides the number of meetings attended by mothers, for example, the number of home visits received since the beginning of intervention. It would have also been desirable to use other indicators related to fidelity of volunteers to the intervention protocols. Another limitation of the study consists that the assessors could not be blind in regards to the allocation of the participants, since it was practically impossible not to recognize when a participant of the study was also a participant of the intervention group. To reduce bias, interviewers had not taken part in the intervention and were trained using proper instruction manual for their questionnaires. Anthropometry assessment followed standardized procedures.

In this study, the provision of the intervention and the adhesion of the beneficiaries have not been controlled to ensure the ideal compliance of the protocols. However, the participation of the team that designed the intervention and its evaluation implies a higher degree of stimulation in the provision of the intervention than there would be in routine conditions in a larger scale. Thus, this could be classified as a public health “program efficacy study” in the typology of evaluation studies proposed by Victora et al. [43] taking into account the level of control exercised on the dose in which the intervention would reach the beneficiaries. We thus have to place the findings of this research in an intermediary level between that of a “regime efficacy study” (in which the impact of the intervention tends to be greater) and that of a “program effectiveness study” in routine conditions (in which the impact of the intervention tends to be smaller). In the pair-matched design, in which a control area with similar socioeconomic conditions was assigned to each geographic area (branch) of intervention, the analysis of about twenty variables, following a hypothetical causal model, showed great homogeneity in the baseline characteristics of both studied groups. The variables in which there were differences were taken into account to build statistical models that allowed the adjustment of the analysis of the intervention effect with regard to possible confounding effects. This brings the design used closer to the benefits that would have a cluster randomized design.

## Conclusions

Despite finding effect measures pointing to effects in the desired direction related to malnutrition, we could only detect a reduction in the risk of overweight attributable to the intervention. The findings related to obesity prevention may be of interest in the context of the nutritional transition. Given the size of this study, the results are encouraging and we believe a larger study is warranted. These results draw attention to the potential of community interventions carried out by lay volunteers, with an integral approach since pregnancy, to achieve important progress in the double task of preventing undernutrition and obesity, as part of the development process in low-income and middle-income countries.

## Competing interests

The authors declare that they have no competing interests.

## Authors’ contributions

JN and DMS conceived the research question and the study design. JJP assisted in the questionnaires development and the initial data analyses. JN performed the statistical analyses and drafted the manuscript. AAF and AB supervised the analysis and interpretation of the findings, as well as the writing of the manuscript. DMS supervised the general process. All authors read and approved the final manuscript.

## Pre-publication history

The pre-publication history for this paper can be accessed here:

http://www.biomedcentral.com/1471-2458/13/212/prepub

## Supplementary Material

Additional file 1 Baseline data according to follow-up status. Click here for file
