# Supplementary material for: The double task of preventing malnutrition and overweight: a quasi-experimental community-based trial
Source: BMC Public Health. 2013 Mar 9;13:212. doi: 10.1186/1471-2458-13-212 (PMC3621778; doi:10.1186/1471-2458-13-212)
Supplement: Additional file 1 — Baseline data according to follow-up status. [file 1471-2458-13-212-S1.docx]

**Baseline data according to follow-up status**

|  | Intervention group | | | Control group | | |
| --- | --- | --- | --- | --- | --- | --- |
| Characteristics | Lost | Analyzed |  | Lost | Analyzed |  |
|  | n= 73 | n= 193 |  | n= 78 | n= 259 |  |
|  | Frequency (%)^a^ | Frequency (%)^a^ | *P* | Frequency (%)^a^ | Frequency (%)^a^ | *P* |
|  |  |  |  |  |  |  |
| Maternal education , years |  |  | 0.442 |  |  | 0.091 |
| 0-5 | 18 (24.66) | 39 (20.31) |  | 23 (30.67) | 54 (21.26) |  |
| ≥ 6 | 55 (75.34) | 153 (79.69) |  | 52 (69.33) | 200 (78.74) |  |
| Household wealth index ^b^ | 47.98 ± 21.12 | 50.54 ± 19.14 | 0.346 | 43.01 ± 16.84 | 49.43 ± 20.55 | 0.012* |
| Household income, minimum wage per month |  |  | 0.469 |  |  | 0.689 |
| 1 | 55 (78.57) | 138 (74.19) |  | 61 (84.72) | 201 (80.40) |  |
| ≥ 2 | 15 (21.43) | 48 (25.81) |  | 11 (15.28) | 49 (19.60) |  |
| Mother had worked during pregnancy ^c^ | 13 (17.81) | 47 (24.35) | 0.254 | 14 (17.95) | 59 (22.78) | 0.825 |
| Permanent economic support from father during pregnanc | 56 (77.78) | 161 (83.42) | 0.289 | 68 (87.18) | 214 (82.63) | 0.340 |
| Mother possessed “Comer es primero” card in 2005 ^d^ | 8 (11.11) | 24 (12.63) | 0.737 | 8 (11.94) | 32 (12.65) | 0.876 |
| National origin of the mother |  |  | 0.685 |  |  | 0.380 |
| Dominican | 67 (91.78) | 174 (90.16) |  | 67 (85.90) | 230 (89.49) |  |
| Haitian | 6 (8.22) | 19 (9.84) |  | 11 (14.10) | 27 (10.51) |  |
| Piped water inside the house (or outside, rural) | 25 (34.25) | 78 (40.41) | 0.357 | 26 (33.33) | 114 (44.02) | 0.093 |
| Waste disposed in a nearby dump, river or glen | 17 (26.15) | 42 (23.33) | 0.648 | 33 (48.53) | 80 (33.90) | 0.028* |
| More than 2 children aged 0-4 in the household | 9 (12.33) | 19 (9.84) | 0.556 | 11 (14.10) | 30 (11.58) | 0.551 |
| Father lived in the house with mother during pregnancy | 52 (71.23) | 150 (77.72) | 0.269 | 63 (80.77) | 199 (77.13) | 0.497 |
| Maternal height, cm ^b^ | 158.70 ± 6.77 | 159.03 ± 6.06 | 0.704 | 158.24 ± 7.80 | 159.12 ± 6.43 | 0.321 |
| Maternal BMI, kg/m^2 e^ | 22.02 ± 1.23 | 22.27 ± 1.20 | 0.697 | 22.59 ± 1.21 | 22.46 ± 1.20 | 0.836 |
| Maternal age, years ^e^ | 21.30 ± 1.28 | 24.08 ± 1.26 | 0.000* | 23.12 ± 1.28 | 23.28 ± 1.26 | 0.823 |
| First born child | 31 (42.47) | 55 (28.50) | 0.030* | 18 (23.08) | 71 (27.41) | 0.446 |
| Desire for pregnancy |  |  | 0.860 |  |  | 0.866 |
| Wanted to become pregnant at the time | 23 (31.51) | 63 (32.64) |  | 26 (33.33) | 89 (34.36) |  |
| Wanted to wait or did not want more children | 50 (68.49) | 130 (67.36) |  | 52 (66.67) | 170 (65.64) |  |

* variables with p<0.05

^a^ The percentages are based on the number of completed responses for each particular variable.

^b^ The values presented are: average ± standard deviation

^c^ Mother made money during pregnancy either through formal or occasional job or even working for herself.

^d^ This is the principal food aid program of the government.

^e^ The presented values are: geometric mean ± geometric standard deviation, because the logarithmic transformation of the variable was necessary.
